# Efficacy and safety of the extracorporeal shockwave therapy in patients with postherpetic neuralgia: study protocol of a randomized controlled trial

**DOI:** 10.1186/s13063-020-04564-z

**Published:** 2020-07-08

**Authors:** Lu Chen, Ruihao Zhou, Fuguo Sun, Yan Weng, Ling Ye, Pingliang Yang

**Affiliations:** 1 Department of Pain Management, West China Hospital, Sichuan University, Guoxuexiang No. 37, Chengdu, 610041 People’s Republic of China; 2 Department of Anesthesiology, The First Affiliated Hospital of Chengdu Medical College, Xindu, 610500 Sichuan People’s Republic of China

**Keywords:** Extracorporeal shockwave therapy, Postherpetic neuralgia, Neuropathic pain, Randomized controlled trial

## Abstract

**Background:**

Postherpetic neuralgia (PHN) is one of the most common types of chronic neuropathic pain, which seriously affects quality of the life because of pain severity and poor response to the currently available treatments. The main strategies for PHN management are medication and invasive interventional therapies; however, these approaches have many adverse effects, so it is important to find another effective and safe treatment for PHN.

**Methods:**

A single-center, single-blind randomized clinical trial will evaluate 98 study participants randomized in a 1:1 ratio into control and experimental groups. The control group will receive conventional treatment including medication therapy and invasive interventional therapy. The experimental group will receive extracorporeal shockwave therapy (ESWT) in addition to conventional therapy. The primary outcome is pain intensity assessed on a visual analogue scale (VAS); the secondary outcomes are the following: quality of life assessed by the 36-Item Short-Form Health Survey (SF-36), psychological state for anxiety and depression measured by the Self-Rating Anxiety Scale (SAS) and Self-Rating Depression Scale (SDS), and sleep quality measured by the Pittsburgh Sleep Quality Index (PSQI). Assessors blinded to the randomization will collect data during the intervention period at baseline and weeks 1, 4, and 12. The plasma levels of tumor necrosis factor-α and interleukin-6 will be assessed before and after ESWT to explore the biochemical mechanisms of ESWT in the treatment of PHN.

**Discussion:**

This randomized controlled trial will evaluate the effectiveness and safety of ESWT in patients with PHN and thus will provide clinical evidence for its use in the management of PHN and explore the potential biochemical mechanisms of this treatment.

**Trial registration:**

www.ChiCTR.org.cn, identifier: ChiCTR1900025828. Registered on 10 September 2019

## Background

Postherpetic neuralgia (PHN) is one of the most common types of chronic neuropathic pain, which is defined as neuropathic pain that persists 3 months or more after the resolution of shingles (herpes zoster) [1]. This neuralgia occurs in nearly 20% of patients with herpes zoster, and the risk increases with patient age, most notably between ages 50 and 79 years [2, 3]. Patients with PHN may experience constant burning, itching, and sharp or lightning pain, and some may experience allodynia or hyperalgesia, which seriously affects the quality of life because of the severity of pain and the poor response to the currently available treatments [4].

The main pain management strategies for patients with PHN include medication and invasive interventional therapies. The first-line drugs include gabapentin, pregabalin, lidocaine patch 5%, and antidepressants [3]. Although medication therapy is effective, a number of adverse effects (e.g., constipation, nausea, and vomiting) often limit its practical use. When medication therapy cannot effectively relieve pain or if adverse effects are intolerable, then invasive interventional therapy may be considered, including nerve block, pulsed radiofrequency therapy (regulation and thermocoagulation), transcutaneous electrical nerve stimulation, and spinal cord stimulation [5]. However, invasive interventional therapy still has risks of infection, bleeding, and nerve injury. Therefore, it is urgent to find an effective and safe treatment for PHN.

A new technique for managing PHN is extracorporeal shockwave therapy (ESWT), which induces single-impulse transient acoustic waves by electromagnetic, electrohydraulic, and piezoelectric devices [6]. During ESWT, a probe is focused on the painful area with the energy and frequency of shockwave adjusting according to the patient’s reaction. Use of ESWT is increasing because it is noninvasive and has low rate of complications compared with other treatments. In addition, some studies have shown potential efficacy for ESWT in neuropathic pain such as Morton’s neuroma, trigeminal neuralgia, and diabetic foot ulcers [7–11]. However, no randomized controlled trials to date have confirmed the efficacy and safety of ESWT in patients with PHN or explored the potential mechanisms of action. Therefore, this randomized controlled trial will evaluate the effectiveness and safety of ESWT in patients with PHN to provide clinical evidence for its use in PHN management.

## Methods

### Study design

**Fig. 1:**
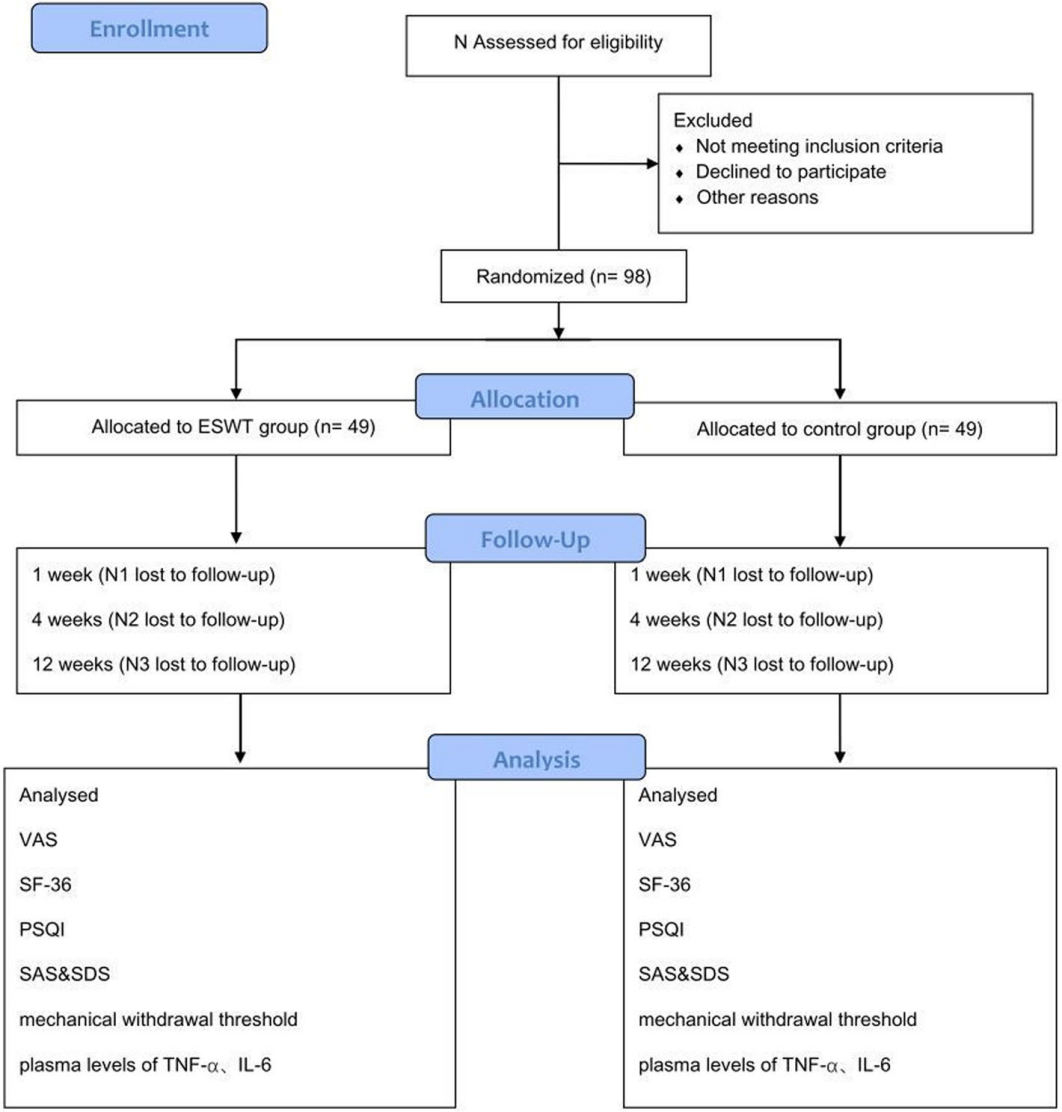
CONSORT flow diagram. ESWT, extracorporeal shockwave therapy; VAS, visual analogue scale; SF-36, 36-Item Short-Form Health Survey; SAS, Self-Rating Anxiety Scale; SDS, Self-Rating Depression Scale; PSQI, Pittsburgh Sleep Quality Index

**Fig. 2:**
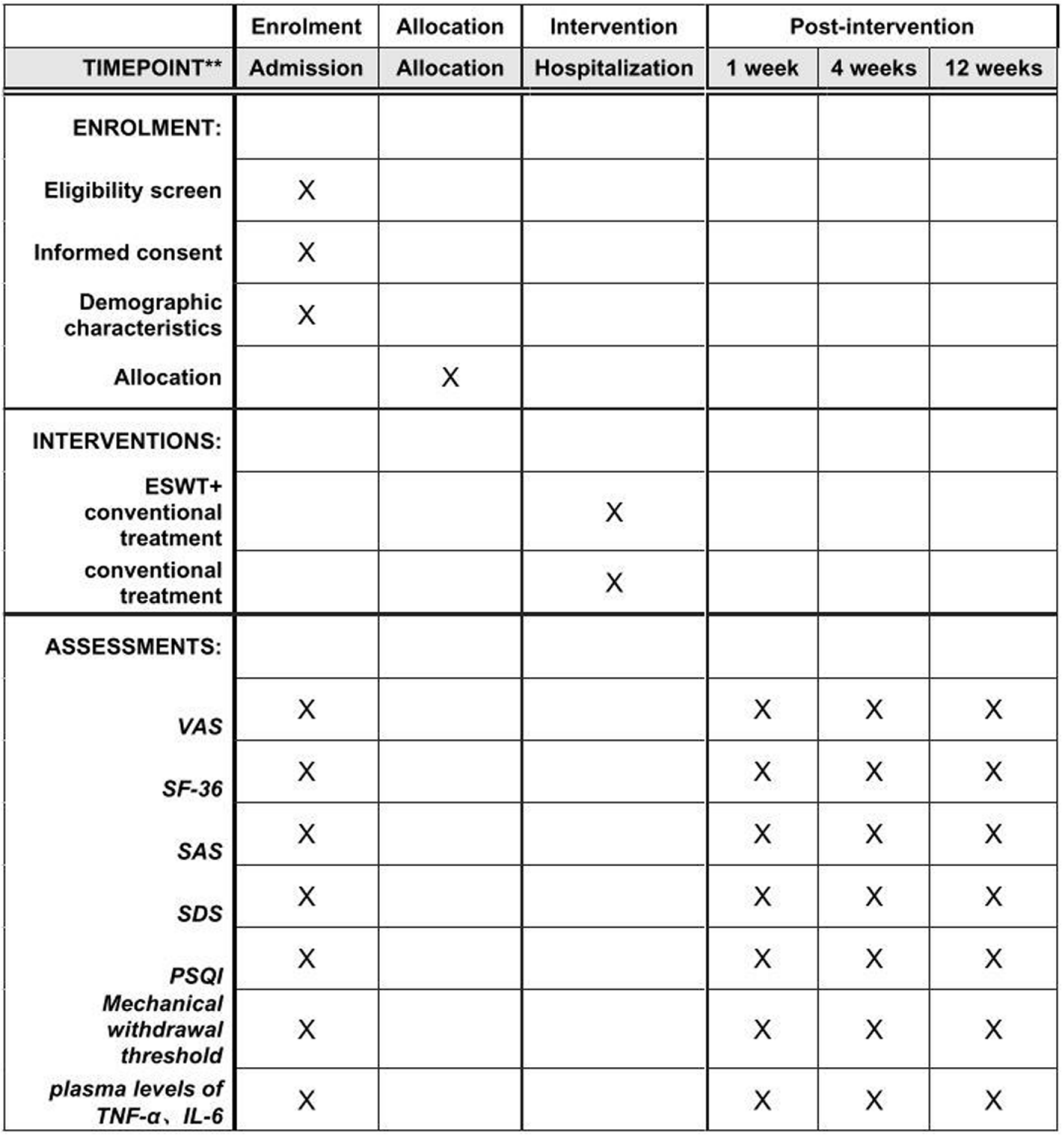
Standard Protocol Items: Recommendation for Interventional Trials (SPIRIT) schedule of enrollment, interventions, and assessments. ESWT, extracorporeal shockwave therapy; VAS, visual analogue scale; SF-36, 36-Item Short-Form Health Survey; SAS, Self-Rating Anxiety Scale; SDS, Self-Rating Depression Scale; PSQI, Pittsburgh Sleep Quality Index

This single-center, single-blind randomized clinical trial was approved by the Ethics Committee of West China Hospital, Sichuan University, Chengdu, China (version 2.0 of this protocol was approved on 30 December 2019, reference 2019[814]), and has been prospectively registered at ChiCTR.org.cn (ChiCTR1900025828) on 10 September 2019. It will be conducted in the Department of Pain Management, West China Hospital, from February 2020 to December 2020. The trial flowchart is shown in Fig. 1. Additional file 1 shows the Standard Protocol Items: Recommendations for Interventional Trials (SPIRIT) checklist. The schedule of enrollment, interventions, and assessments follows the SPIRIT checklist (Fig. 2).

A total of 98 participants will be randomized into a control group or an experimental group. Assessors blinded to the randomization will collect data during the intervention period at baseline and study weeks 1, 4, and 12. The plasma levels of tumor necrosis factor-α (TNF-α) and interleukin (IL)-6 will be detected before and after ESWT to explore the biochemical mechanisms of ESWT for the treatment of PHN.

### Selection of the participants

Patients diagnosed with PHN according to the European Consensus-Based (S2k) Guideline on the Management of Herpes Zoster will be informed of this trial [12]. Potential participants will be identified according to their medical record at the time of hospital admission. After a brief introduction about the trial, patients may decide whether they will participate in this study. Then, detailed information will be acquired to assess patient eligibility according to the following criteria (Table 1). All participants must sign the informed consent form before treatment. In the consent form, participants will be asked if they agree to use of their data after withdrawing from the trial. Participants will also be asked for the permission to share relevant data with university researchers and regulatory authorities. Participants will be informed that this trial does not involve collecting biologic specimens for the storage. Two physicians in the Department of Pain Management will approach the patients about taking part in the trial, enroll participants, and obtain their informed consent.

**Table 1:** Eligibility criteria

Inclusion criteria	Exclusion criteria
▪ Diagnosed with PHN	▪ Allergic to medical ultrasonic couplant
▪ VAS ≥ 4 points	▪ History of tumor
▪ Age ≥ 18 years	▪ Liver or kidney dysfunction
▪ Describe symptoms clearly and have ability to self-evaluate	▪ Schizophrenia or dementia
	▪ Cannot be followed up on schedule
▪ Did not previously receive ESWT	▪ History of thrombosis
▪ Did not participate in other clinical trials within past 3 months	▪ Disturbance coagulation function using anticoagulants
	▪ Cardiac pacemaker use
	▪ Infection in the pain area
	▪ Pregnant or puerperal patients
	▪ Fracture or severe osteoporosis

*Abbreviations*: *ESWT* extracorporeal shockwave therapy, *PHN* postherpetic neuralgia, *VAS* visual analogue scale

### Sample size

The sample size was estimated based on a previous study that used effective rates as the primary outcome (experimental group, 96%; control group, 77%) [13]. Power analysis software (G*Power, version 3.1.9.4) was used with the superiority test (one-tailed test [*α*, 0.05; *ß*, 0.2]) and estimated a sample size of 78 participants (39 participants per group). Considering potential dropout rate of 20%, a total of 98 participants will be enrolled in this study.

### Patient and public involvement

There were no funds or time allocated for patient and public involvement, so we were unable to involve patients. We have invited patients to help us develop our dissemination strategy.

### Randomization and blinding

Patients will be randomized into a control group or an experimental group in a 1:1 ratio based on random numbers generated by Excel spreadsheet. Sealed opaque envelopes were used for allocation concealment. A researcher will generate the allocation sequence, and another researcher will assign participants to the control or experimental group. The assessors and statisticians were blinded to randomization and did not participate in the treatment.

### Interventions

#### Control group

Patients in the control group will receive conventional treatment including medication therapy and invasive interventional therapy.

##### Medication therapy

1. Anticonvulsion medicine

Gabapentin: starting dose of 0.3 g on day 1, 0.6 g on day 2, and 0.9 g on day 3, then a maintenance dose of 0.9 g/day, adjusted based on the patient’s reaction.

Pregabalin: starting dose of 75 mg twice a day, then adjusted based on the patient’s reaction.

2. Opioid analgesics

Oxycodone and acetaminophen: 1 tablet 3 times a day, adjusted based on the patient’s pain intensity.

3. Neural nutrients

Mecobalamin: 0.5 mg three times a day.

##### Invasive interventional therapy

Pulsed radiofrequency regulation guided by low-dose computed tomography will be administered based on the patient’s condition.

#### Experimental group

The experimental group will be treated with ESWT in addition to conventional therapy. The patient may be placed in the prone, lateral, or seated position depending on the painful area. Treatment with ESWT will be performed with a radial extracorporeal shockwave generator (MASTERPULS MP100; Storz Medical AG, Tägerwilen, Switzerland), and all treatments will be performed by the same therapist who is formally trained in ESWT. After applying the ultrasonographic coupling agent to the skin of treated area, the patients will receive 5000 to 7000 pulses every session by a R15 probe (radius of 15 mm) at a frequency of 10 Hz, with energy gradually increasing from 1 to 4 bar depending on the patient’s reaction. The therapist will move the probe in transverse or longitudinal directions along the nerve. The procedure will be performed every 3 to 5 days, and a total of 3 to 5 sessions constitute a therapeutic course.

### Adherence monitoring

Patients or their family members will be asked to report the actual drug intake to ensure treatment compliance. In addition, the therapist will gradually increase the energy of shockwave from 1 to 4 bar and adjust the energy according to the patient’s reaction to improve adherence to the intervention.

### Outcome measure

#### General conditions

The patient’s age, gender, body mass index, previous medical history, duration of PHN, area of nerve pain, medication use, and previous treatments will be obtained at baseline.

#### Primary outcome

The visual analogue scale (VAS) will be used to evaluate pain intensity at baseline and at study weeks 1, 4, and 12. This scale is a 10-cm horizontal line with terminal descriptors of 0 (no pain) and 10 (worst imaginable pain) [14]. The participants will mark the line position according to their degree of pain, and the VAS score is determined by measuring the distance from 0 to the marked point.

#### Secondary outcome

Secondary outcomes will be collected at baseline and at study weeks 1, 4, and 12.

1. Quality of life will be accessed by 36-Item Short-Form Health Survey (SF-36) which measures three aspects of health: functional ability, well-being, and overall health [15].
2. Anxiety and depression are common mental disorders in patients with chronic pain [16]. The psychological state of patients will be measured by the Self-Rating Anxiety Scale (SAS) and the Self-Rating Depression Scale (SDS).
3. Sleep quality will be assessed by the Pittsburgh Sleep Quality Index (PSQI), which comprises 19 questions and 7 sleep components: perceived sleep quality, sleep latency, sleep duration, habitual sleep efficiency, sleep disturbances, sleep medications, and daytime dysfunction [17].
4. Mechanical withdrawal threshold will be tested using von Frey hairs (Aesthesiometer, Somedic, Sweden) according to the methods used by Moharić et al. [18] to indicate the level of peripheral sensitization.
5. Plasma levels of TNF-α and IL-6 will be detected before and after ESWT to explore the biochemical mechanisms of ESWT for the treatment of PHN.

The researchers will provide medical advice to participants who complete follow-up to promote participant retention. Participants who discontinue or deviate from intervention protocols will be recorded in the case report form, and their data, including outcome, adverse event, and the reason of dropping out, will be analyzed for further discussion.

### Safety evaluation

Adverse effects will be recorded, including petechiae and swelling of the skin, allergy, fever, paresthesia, pain aggravation, tissue edema, and any other reported adverse reactions to ESWT. In addition, incidence of adverse effects will be analyzed as a measurement of safety. Serious adverse events are described as events that prolong hospitalization time, cause disability, affect ability to work, or endanger life in clinical trials. If a serious adverse event occurs, treatment will be suspended immediately and remedy actions taken appropriately and freely in time. All serious adverse events will be reported to the Ethics Committee and relevant responsible research units to determine whether to stop the clinical trial.

### Monitoring

An Independent Data Monitoring Committee (IDMC), which is separate from the trial sponsor and competing interests, will supervise the trial including assessment of the progress, safety data, and efficacy endpoints. The trial sponsor may decide whether to continue, modify, or stop the trial according to the recommendations of the IDMC. The Project Management Group will review the trial conduct monthly. The Trial Steering Group and the IDMC will review the trial conduct every 3 months, and the Ethics Committee will review the trial conduct annually.

### Data management and statistical analysis

All original data will be stored in a case report form, and any personal information will be protected. The study supervisor will have access to the trial dataset and make the final decision to terminate the trial. Audits of the trial will be performed bimonthly. Data analysis will be performed by SPSS software (version 22; SPSS Inc., Chicago, IL, USA). Continuous data will be presented as mean ± standard deviation, and categorical variables will be reported as numbers or percentages. The basic characteristics of participants will be described and compared by the Student *t* test to ensure comparability. All statistical analysis followed the principle of intent-to-treat analysis. The difference in treatment effect between the two groups will be evaluated by one-way analysis of variance (ANOVA) with repeated measures ANOVA to analyze the changes over time in the intervention group. The duration of PHN and painful segment, as potential factors, will be assessed using the logistic regression. All tests will be single-sided, and *p* < 0.05 will be considered statistically significant. All statistical analysis will follow the principle of intent-to-treat analysis. The patients randomized to the intervention group but who do not adhere to the intervention will be included in the final data analysis. Imputation method will be used to handle missing data if the assumption of missing at random is met.

## Discussion

As a common condition in older adults who have had shingles, PHN seriously affects quality of life and mental health. Although multiple therapies for PHN are available, a recommended effective and safe treatment is still lacking. The aim of this randomized controlled trial is to assess the efficacy and safety of ESWT in patients with PHN and explore the potential biochemical mechanisms of this treatment.

Persistent inflammation, oxidative stress-mediated injury, and cytokine activation are shown to play an important role in the pathological mechanism of neuropathic pain [7]. Some studies suggest that TNF-α, IL-1, IL-6, and IL-17 may be the main pro-inflammatory cytokines in neuropathic pain [19]. To the knowledge of the researchers for this proposed trial, the mechanism of ESWT for PHN may be related to immune regulation and analgesic effect. A recent study showed that ESWT may reduce the plasma levels of TNF-α and IL-6, which had an anti-inflammatory effect [20]. In addition, decreased substance P levels after ESWT may produce analgesic effects [21]. Hopefully, the results of the proposed study will confirm the effect of ESWT for patients with PHN and provide clinical evidence to support its use. Finally, if the study hypothesis is verified, ESWT may be considered for recommendation as an effective and safe therapy for PHN.

The proposed study has several limitations. First, it is a single-center clinical trial, which might influence participant recruitment. Second, this clinical trial is single-blind because a double-blind design would be difficult to ensure considering the therapeutic properties of ESWT, which might cause patient bias. In addition, the primary outcome measure is a subjective evaluation, which may be a potential risk for bias because participants will know in which arm of the trial they are enrolled. Another limitation is that although the trial follow-up is 3 months after the treatment, extended follow-up may be needed to demonstrate the benefits of ESWT in further studies.

## Trial status

The protocol version is 2.0 (issue date: 29 October 2019). Recruitment began in February 2020 and will probably be completed at the end of May 2020.

## Supplementary information

**Additional file 1 Standard Protocol Items**: Recommendations for Interventional Trials (SPIRIT) Checklist for the protocol of a clinical trial.

## Data Availability

The datasets analyzed during the current study are available from the corresponding author on reasonable request.

## Abbreviations

ANOVA: Analysis of variance
ESWT: Extracorporeal shockwave therapy
IDMC: Independent Data Monitoring Committee
PSQI: Pittsburgh Sleep Quality Index
SAS: Self-Rating Anxiety Scale
SDS: Self-Rating Depression Scale
VAS: Visual analogue scale

## References

[1] Hadley GR, Gayle JA, Ripoll J, Jones MR, Argoff CE, Kaye RJ (2016). Post-herpetic neuralgia: a review. Curr Pain Headache Rep.

[2] Forbes HJ, Bhaskaran K, Thomas SL, Smeeth L, Clayton T, Mansfield K (2016). Quantification of risk factors for postherpetic neuralgia in herpes zoster patients: a cohort study. Neurology..

[3] Fashner J, Bell AL (2011). Herpes zoster and postherpetic neuralgia: prevention and management. Am Fam Physician.

[4] Forbes HJ, Thomas SL, Smeeth L, Clayton T, Farmer R, Bhaskaran K (2016). A systematic review and meta-analysis of risk factors for postherpetic neuralgia. Pain.

[5] Lin CS, Lin YC, Lao HC, Chen CC (2019). Interventional treatments for postherpetic neuralgia: a systematic review. Pain Phys.

[6] Ji Q, Wang P, He C (2016). Extracorporeal shockwave therapy as a novel and potential treatment for degenerative cartilage and bone disease: osteoarthritis. A qualitative analysis of the literature. Prog Biophys Mol Biol.

[7] Chen KH, Yang CH, Wallace CG, Lin CR, Liu CK, Yin TC (2017). Combination therapy with extracorporeal shock wave and melatonin markedly attenuated neuropathic pain in rat. Am J Transl Res.

[8] Yang CH, Yip HK, Chen HF, Yin TC, Chiang JY, Sung PH (2019). Long-term therapeutic effects of extracorporeal shock wave-assisted melatonin therapy on mononeuropathic pain in rats. Neurochem Res.

[9] Seok H, Kim SH, Lee SY, Park SW (2016). Extracorporeal shockwave therapy in patients with Morton’s neuroma a randomized, placebo-controlled trial. J Am Podiatr Med Assoc.

[10] Zhang D, Meng Y, Hai H, Yu XT, Ma YW (2018). Radial extracorporeal shock wave therapy in an individual with primary trigeminal neuralgia: a case report and literature review. Am J Phys Med Rehabil.

[11] Galiano R, Snyder R, Mayer P, Rogers LC, Alvarez O (2019). Focused shockwave therapy in diabetic foot ulcers: secondary endpoints of two multicentre randomised controlled trials. J Wound Care.

[12] Werner RN, Nikkels AF, Marinovic B, Schafer M, Czarnecka-Operacz M, Agius AM (2017). European consensus-based (S2k) guideline on the management of Herpes Zoster - guided by the European Dermatology Forum (EDF) in cooperation with the European Academy of Dermatology and Venereology (EADV), part 1: diagnosis. J Eur Acad Dermatol Venereol.

[13] Yang J, Hu H, Huang Z (2019). Effect of neurotropin combined with extracorporeal shock wave therapy in the treatment of postherpetic neuralgia. Chinese Community Doctors.

[14] Hawker GA, Mian S, Kendzerska T, French M (2011). Measures of adult pain: Visual Analog Scale for Pain (VAS Pain), Numeric Rating Scale for Pain (NRS Pain), McGill Pain Questionnaire (MPQ), Short-Form McGill Pain Questionnaire (SF-MPQ), Chronic Pain Grade Scale (CPGS), Short Form-36 Bodily Pain Scale (SF-36 BPS), and Measure of Intermittent and Constant Osteoarthritis Pain (ICOAP). Arthritis Care Res.

[15] Patel AA, Donegan D, Albert T (2007). The 36-item short form. J Am Acad Orthop Surg.

[16] Rus Makovec M, Vintar N, Makovec S (2015). Self-reported depression, anxiety and evaluation of own pain in clinical sample of patients with different location of chronic pain. Zdr Varst.

[17] Morris JL, Rohay J, Chasens ER (2018). Sex differences in the psychometric properties of the Pittsburgh Sleep Quality Index. J Women's Health (Larchmt).

[18] Moharic M, Vidmar G, Burger H (2012). Sensitivity and specificity of von Frey’s hairs for the diagnosis of peripheral neuropathy in patients with type 2 diabetes mellitus. J Diabetes Complicat.

[19] Hung AL, Lim M, Doshi TL (2017). Targeting cytokines for treatment of neuropathic pain. Scand J Pain.

[20] Wang CJ, Huang CC, Yip HK, Yang YJ (2016). Dosage effects of extracorporeal shockwave therapy in early hip necrosis. Int J Surg.

[21] Maier M, Averbeck B, Milz S, Refior HJ, Schmitz C (2003). Substance P and prostaglandin E2 release after shock wave application to the rabbit femur. Clin Orthop Relat Res.

